# Antibacterial and Antiproliferative Activities of *Azadirachta indica* Leaf Extract and Its Effect on Oil-in-Water Food Emulsion Stability

**DOI:** 10.3390/molecules27227772

**Published:** 2022-11-11

**Authors:** Manel Ouerfelli, Isidoro Metón, Idoia Codina-Torrella, María Pilar Almajano

**Affiliations:** 1 Chemical Engineering Department, Escola Tècnica Superior d’Enginyeria Industrial de Barcelona (ETSEIB), Universitat Politècnica de Catalunya, Av. Diagonal 647, 08028 Barcelona, Spain; 2 Biology Department, Faculty of Sciences of Tunis, University of Tunis El Manar, Tunis 2092, Tunisia; 3 Biochemistry and Physiology Departament, Facultat de Farmàcia i Ciències de l’Alimentació, Universitat de Barcelona, Joan XXIII 27-31, 08028 Barcelona, Spain; 4 Agri-Food Engineering and Biotechnology Department, Escola d’Enginyeria Agroalimentària i de Biosistemes de Bacelona (EEABB), Universitat Politècnica de Catalunya, Esteve Terrades, 8, 08860 Castelldefels, Spain

**Keywords:** antioxidant, *Azadirachta indica*, anti-bacterial, cancer cells, food emulsion, HPLC-MS

## Abstract

The present study aims to identify and quantify the phenolic compounds of *Azadirachta indica* leaf extract using HPLC-MS and to evaluate the antioxidant, antibacterial (against different Gram-positive and negative bacteria) and in vitro anti-proliferative activities of this extract (against breast, human liver and cervix adenocarcinoma-derived cells). The application of this extract as a natural antioxidant for food preservation was also tested on oil-in-water food emulsions for the first time in the present work in order to determine the use of *Azadirachta indica* leaves as a natural additive to preserve the food against lipid oxidation and rancidity. The results obtained revealed that 50%-aqueous ethanol leaf extract showed the best extraction yield (25.14%), which was characterized by a high content in phenolic compounds and strong antioxidant activity. Moreover, this leaf extract inhibited the growth of the bacterial strains tested (*Staphylococcus aureus, Escherichia coli, Salmonella paratyphi* and *Micrococcus luteus*) and showed better anti-proliferative activity against breast and cervix adenocarcinoma-derived cells than human liver cancer cells after 48 h of treatment. Additionally, *Azadirachta indica* leaf extract showed almost similar effects as gallic acid solutions (0.25% and 0.5%) in preserving the oxidation of oil-in-water food emulsions and prevented the formation of secondary oxidation products (malondialdehyde) as well. The results obtained suggested that extracts of *Azadirachta indica* leaves are a potential source of antioxidant and antibacterial compounds and pointed to the potential of these natural extracts as therapeutic agents.

## 1. Introduction

The oxidation and rapid deterioration of lipids in food present great problems for the food industry [1]. Reactions between fatty acids and oxygen not only occur during the production of foods through to their consumption, but also during the shelf-life of stored products [2]. These oxidative degradation reactions of lipids lead to the formation of different toxic compounds (for example, carbonyls, aldehydes and trienes), as well as to the modification of some of their characteristics and properties, such as changes in their colour and texture, the production of inappropriate odours and flavours or a reduction in their nutritional value [3]. A wide range of food products, such as vegetable oil and food emulsions (milk, cream, mayonnaise, margarine, etc.), contain very high amounts of fats, which vary from one product to another depending on their type [4] and are easily exposed to deterioration during processing and preservation steps, highlighting the importance of studying these products to find effective preservative solutions and extend their shelf life [5].

The addition of antioxidants into food products is one of the most widely used strategies to prevent oxidation reactions to such food products. Synthetic additives, such as butylated hydroxytoluene (BHT), tertiary butylhydroquinone (TBHQ) or butylated hydroxyanisole (BHA), among others, have been successfully used to preserve food systems from oxidation and improve their safety, freshness, taste, texture and appearance [1]. Although synthetic antioxidants are highly effective and produced at low cost, only a few of them are used by the food industry, due to the adverse human health effects associated with their toxicity, which includes the carcinogenic activity of their oxidized forms or other effects such as allergies, headache, dermatitis, etc. [6,7]. For this reason, many countries have adopted strict regulations regarding their use [8]. For instance, previous research studies proved that high doses of BHA and BHT may affect the nervous system and can produce allergic reactions and inflammations in the skin, such as eczema and dermatitis. Consequently, and given the preferential use of natural products by consumers, the search for natural antioxidants from plants to be used as additives in food products has caught the interest of researchers and food industries [9].

Phenolic compounds, such as phenolic acids, flavonoids, terpenoids and carotenoids, have generated a growing interest due to their antioxidant activity, allowing them to preserve food products against rancidity [10]. However, a small number of plants are exploited for their bioactive substances in food processing and preservation. For this reason, several studies have been carried out in the search for active compounds from different parts of plants to benefit from their antioxidant properties in order to maintain the quality of food products, and their applications in the food industry are also progressively addressed [11]. Previous studies have shown that using natural additives from plants clearly delays the lipid peroxidation process, which makes them a good alternative to synthetic additives [12,13]. For example, it has been proved that polyphenols prevent food emulsion from oxidizing [14]. In addition to their antioxidant power, phenolic compounds are commonly known for their excellent therapeutic potential against disease agents, such as fungi, bacteria and viruses [15] and several chronic illnesses such as cardiovascular and neurodegenerative diseases, as well as cancer [16]. Over the past years, plants have been the largest source of metabolites, with the advantage of having excellent anticancer activities [17]. Phenolic compounds, mainly phenolic acids, are associated with potent anticancer abilities in various in vitro and in vivo studies [18]. In addition, the anticancer effects of polyphenolic compounds such as quercetin, curcumin, resveratrol and kaempferol have been investigated on numerous cancer cell lines and shown prominent results [19].

*Azadirachta indica* A. Juss. (*A. indica*) is one of the most useful plants in traditional medicine in Indian culture owing to its therapeutic benefits and high phenolic content [20]. The documented medicinal virtues of *A. indica* showed that its different parts (leaves, flowers, seeds, fruits, roots and barks) have been used to treat several human diseases, such as inflammation, diarrhoea, bacterial infection, constipation [21], cancer [22], fever and skin diseases, among others [23]. Furthermore, *A. indica* has extensive pharmacological activities due to its complex composition, which is characterized by more than 300 different bioactive compounds with multidirectional activities [24]. However, its use as a natural food antioxidant is still restricted. It is necessary to demonstrate the potential and effectiveness of active constituents of *A. indica* extracts to be used as substitutes of synthetic additives, while guaranteeing the health of consumers. 

In this context, the objectives of the present study were, first, to determinate the appropriate conditions for obtaining the *A. indica* leaf extract and to quantify the different phenolic compounds of this sample, in order to evaluate its antioxidant capacity and also to assess its antioxidant effect against lipid oxidation in oil-in-water food emulsions. Thus, this study also aims to evaluate the antibacterial potential and anti-proliferative activity of *A. indica* leaf extract against several cancer cell lines.

## 2. Results and Discussion

### 2.1. Extraction Yield of A. indica EtOH Extracts

Table 1 shows the extraction yield values obtained in *A. indica* leaf extracts.

The results obtained showed that the best extraction yield of *A. indica* leaf extract was observed with the 50%-aqueous EtOH solvent, whereby the extraction yield was 25.14% compared to leaves extracted with 80%-aqueous EtOH that extracted only 19.04%, and absolute EtOH, which extracted less than half as much. 

The choice of the extraction solvent is a very important step to isolate and recover the maximum phytochemical compounds from plant materials [25]. In the present study, ethanol polarity was influenced by a high concentration of water. The addition of milliQ-water to EtOH remarkably increased the values of the extraction yield, which reveals the effectiveness of mixed solvents in improving the extraction of particular phenolic compounds. This circumstance may be explained by the fact that the solubility of phenolic compounds depends on the polarity and the properties of the extraction solvent [26]. Some phenolic compounds are better extracted in inorganic solvents such as water than in organic solvents such as alcohols. Other polyphenols are most soluble in organic solvents less polar than water [27]. Thus, the mixing of water with an organic solvent increases the extraction efficiency of some target compounds that are soluble in both solvents. This fact can be also attributed to the milliQ-water, since it can weaken the strength of the solute–matrix interactions due to the permeability of the cell tissue, allowing for a better transfer of mass by molecular diffusion [26]. Additionally, swelling of the plant material caused by the adsorption of solvent molecules on specific functional groups of biomass components could also be involved [28]. 

### 2.2. Phenolic Compound Content, Antioxidant and Antiradical Activity of A. indica 50% EtOH Extract

The phenolic compound contents varied in *A. indica* leaf extract and exhibited antioxidant activity (Table 2). 

The determination of the chemical composition of *A. indica* 50%-aqueous EtOH extract showed that the contents of the different phenolic compounds were significantly different α = 0.05. TPC was estimated at 47.47 mg GAE/g lyophilized sample, and TFC was approximately 1.4-fold higher than the TCTC. 

The existence of different types of phenolic compounds in *A. indica* extracts was previously reported in other studies. For instance, compared to the results obtained in the present work, Shewale and Rathod [29] reported lower TPC value estimated at 6.64 mg GAE/g dry weight in Indian *A. indica* leaf extracted in 50% EtOH by stirring extraction method and using the standard gallic acid to construct the calibration curve. 

Concerning results obtained in the current study and presented in Table 2, 50%-aqueous EtOH leaf extract has potent antioxidant and antiradical activities. As shown, *A. indica* 50%-aqueous EtOH extract was able to reduce ferric iron (Fe^3+^) to ferrous iron (Fe^2+^) and presented a ferric-reducing ability of 2.30 mM TE/g lyophilized sample, as shown in the FRAP assay. Results obtained by TEAC and ORAC assays also confirmed the antioxidant potential of *A. indica* leaf extract, in which 1.68 and 1.66 mM TE/g lyophilized sample were obtained, respectively. As shown in Table 2, extract of *A. indica* leaves also appeared to be an inhibitor of DPPH radicals, with a radical-scavenging activity equal to 0.37 mM TE/g lyophilized sample. 

### 2.3. Phenolic Profile of A. indica 50% EtOH Extract Determined by HPLC-MS

As shown in Table 3, eleven phenolic compounds were identified in *A. indica* leaf extract in HPLC-MS analysis, of which six corresponded to phenolic acids and five to flavonoids. 

The first nine compounds (no. 1–9) were identified by commercial standards, and the rest of the compounds were characterized by comparing their chromatographic behaviour and *m*/*z* with literature-based data, as shown in Table 3. The six phenolic acids detected in *A. indica* leaf extract were identified using a negative mode of ionization. The compound no. 1 with [M − H]^−^ at *m*/*z* 353.0878 and 353.0880 was characterized as chlorogenic acid. The compound no. 2 with [M − H]^−^ at *m*/*z* 179.0345 and 179.0350 was identified as a caffeic acid. The compound no. 3 with [M − H]^−^ at *m*/*z* 198.05282 and 197.0453 was represented as syringic acid. The compound no. 5 with [M − H]^−^ at *m*/*z* 163.0401 and 163.0393 was identified as p-coumaric acid. The compound no. 6 with [M − H]^−^ at *m*/*z* 193.0506 and 193.0502 was represented as ferulic acid, and the compound no. 7 with [M − H]^−^ at *m*/*z* 223.0612 and 223.0603 was identified as sinapinic acid. Only the compound no. 8 was detected in *A. indica* leaf extract as a flavonoid using a positive mode of ionization with [M + H]^+^ at *m*/*z* 319.0449 and 319.0427and identified as myricetin. The rest of the compounds were represented as flavonoids using a negative mode of ionization, mainly, compound no. 4 was identified as (-)-epicatechin at *m*/*z* 289.0717 and 289.0717, compound no. 9 was assigned as quercetin at *m*/*z* 301.0354 and 301.0375, compound no. 10 was identified as luteolin C-hexoside I at *m*/*z* 448.1006 and 447.0935 and compound no. 11 was characterized as cyanidin 3-O-galactoside at *m*/*z* 448.1011 and 448.0982. 

These results showed that *A. indica* leaf extract was rich in phenolic acids, with contents ranging between 102.209 µg/g lyophilized sample (syringic acid) and 7699.18 (sinapinic acid) µg/g lyophilized sample, whereas the contents of flavonoids were lower and estimated between 1154.11 µg/g lyophilized sample (quercetin) and 4382.05 (myricetin) µg/g lyophilized sample. 

There are many research studies involving phenolic compounds, mainly phenolic acids and flavonoids, due to their various physiological properties, such as anti-allergic, anti-inflammatory, antimicrobial, antiviral, antibacterial, anti-carcinogenic, antithrombotic, cardio-protective and vasodilator activities [34]. The beneficial effects of polyphenols are of particular interest in the food industry. Following research studies of the positive impact of polyphenol consumption on health and the prevention of disease, manufacturers are now marketing polyphenol-enriched foods and dietary supplements [35]. In addition, their antioxidant activity ensures better preservation of food products by preventing lipid peroxidation. In the present study, the order of antioxidant activity relative to Trolox was FRAP > TEAC > ORAC > DPPH. The order of activity reflects the fact that the major components of the phenolic extract are sinapinic acid, caffeic acid, p-coumaric acid, myricetin, luteolin -C-hexoside and cyanidin 3-O-galactoside. The latter three compounds have reducing and metal-chelating activities due to the presence of o-dihydroxy phenol substituents, whereas Trolox does not have this structure. In addition, myricetin has the metal-chelating effect of the flavonol structure with the carbonyl group in the C ring, forming the chelate ring structure with the phenolic group in the A ring. Hence, the FRAP value is high. The ORAC value is more dependent on radical scavenging, so the value is closer to 1 mM TE/g lyophilized sample. The DPPH values are also strongly dependent on radical scavenging, but the radical is a nitrogen one, and nitrogen is more electronegative than carbon, so scavenging is reduced by oxygen-containing substituents close to the phenolic hydroxyl group due to the repulsion between the electronegative atoms. Phenolic acids are one of the main classes of plant phenolic compounds and possess a range of biological properties [36]. For example, ferulic acid is considered one of the most common phenolic compounds with multiple biological and pharmaceutical properties, such as antioxidant, anti-inflammatory, antimicrobial, and anticancer activities [37]. Sinapic acid also showed a potent effect in various pathological conditions such as infections [38], inflammation [39], cancer [40], diabetes [41] and neuro-degeneration [42]. The antioxidant power of caffeic acid has also been confirmed by radical scavenging studies [43]. In addition, chlorogenic acid is one of the most important phenolic acids, with several therapeutic effects, such as antioxidant activity, as well as antibacterial, hepato-protective, cardio- protective, anti-inflammatory, antipyretic, neuro-protective, anti-obesity, antiviral, anti- microbial and anti-hypertension activities [44].

Likewise, flavonoids have strong biological and therapeutic effects. For instance, quercetin is known for its antioxidant, anti-inflammatory, antibacterial, antiviral, radical scavenging, gastro-protective and immune-modulatory activities [45]. In addition, myricetin is one of the key ingredients of various foods and beverages well-recognized for its nutraceutical value and strong antioxidant, anticancer, anti-diabetic and anti-inflammatory activities [46]. Furthermore, recent research reported that a high consumption of epicatechin is associated with a decreased risk of cardiovascular mortality [47]. All these phenolic compounds constitute useful elements for the treatment and prevention of human disease, but they also strongly participate in the preservation of the quality of food products.

### 2.4. Oxidative Stability of O/W Emulsion with A. indica 50% EtOH Leaf Extract

#### 2.4.1. Primary Oxidation Products (Peroxide Value)

**Figure 1:**
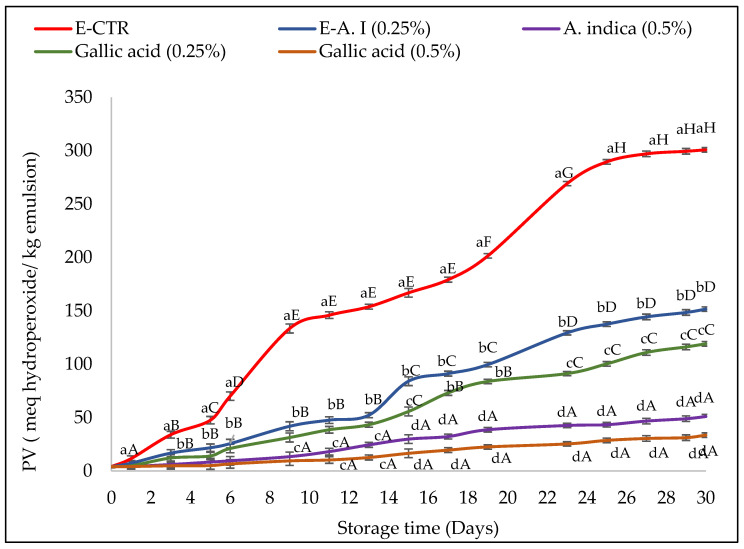
Peroxide value (PV) of emulsions incorporated with *A. indica* leaf 50%-aqueous EtOH extract (0.25% and 0.5%, *v*/*v*) during storage. Error bars represent the standard deviation (n = 3). Lowercase letters indicate significant differences between emulsion samples on the same day at α = 0.05, and uppercase letters indicate significant differences between the days of storage for the same sample at α = 0.05.

In this study, the oxidative stability of emulsions (with 0.25% and 0.5% (*v*/*v*) of *A. indica* leaf 50%-aqueous extract and gallic acid) was evaluated according to the determination of the peroxide value (PV) evolution of samples during their storage. The results obtained are shown in Figure 1.

Considering that the maximum PV estimated by the Codex Alimentarius for an edible refined oil corresponds to 10 meq hydroperoxides/kg of oil, the formation of hydroperoxides was significantly faster in the E-CTR sample, which showed an increase in the primary oxidation values from the first day of storage. The E-GA (0.25%, *v*/*v*) and E-AI (0.25%, *v*/*v*) samples remained stable against lipid oxidation for six days and then exhibited a higher degree of oxidation, whereas E-GA (0.5%, *v*/*v*) and E-AI (0.5%, *v*/*v*) samples were stable against lipid oxidation and exceeded 10 meq hydroperoxides/kg of oil only after nine days of storage. 

At the end of the storage period (30 days), the E-GA (0.25%, *v*/*v*) and E-AI (0.25%, *v*/*v*) samples deteriorated with maximum PV of 119.14 and 151.63 meq hydroperoxides/kg emulsion, respectively. The E-GA (0.5%, *v*/*v*) and E-AI (0.5%, *v*/*v*) samples presented the best protective effect against the formation of the primary oxidation products with values estimated at 33.58 and 51.02 meq hydroperoxides/kg emulsion, respectively, at the end of the storage period.

The *A. indica* leaf extract used in the present study was also effective at retarding lipid oxidation in oil-in-water emulsions. The ability of *A. indica* leaf extract to maintain oxidative stability of the emulsion is probably due to the potent antioxidant capacity of its phenolic compounds, previously demonstrated, which causes deactivation of ROS and interruption of the radical propagation chain reaction, as well as chelation of transition metals, such as Fe and Cu, which catalyse lipid oxidation, as well as reduction in the partial pressure of oxygen [48,49]. In the literature, other authors have also reported the potential of different plant extracts as promising sources of natural antioxidants for improving lipid oxidation stability of oil-in-water emulsions. For example, Gallego et al. [50] reported that lyophilized extracts of *Caesalpinia decapetala* (at concentrations of 0.002 to 0.2%) in oil-in-water emulsions (1% tween-20 and 10% purified sunflower oil) enhanced lipid peroxidation during the storage of the samples (30 days, ~33 °C). In their studies, the concentration of plant extract directly impacted the improvement of the oxidative deterioration of the samples. Skowyra et al. [51] found that *P. frutescens* extracts had good antioxidant properties in 10% sunflower oil-in-water emulsions during storage at 32 °C and were as effective as the synthetic preservative (BHA). Mohd Azman et al. [52] also showed that *Gentiana Lutea root extract* exhibited a synergic effect and better antioxidant activity in delaying lipid oxidation of oil-in-water emulsions (1% Tween-20 and 10% oil) if working with 0.5% *Gentiana Lutea* extract (*w*/*w*) with 0.1% (*w*/*w*) of Bovine Serum Albumin. 

#### 2.4.2. pH Changes

**Figure 2:**
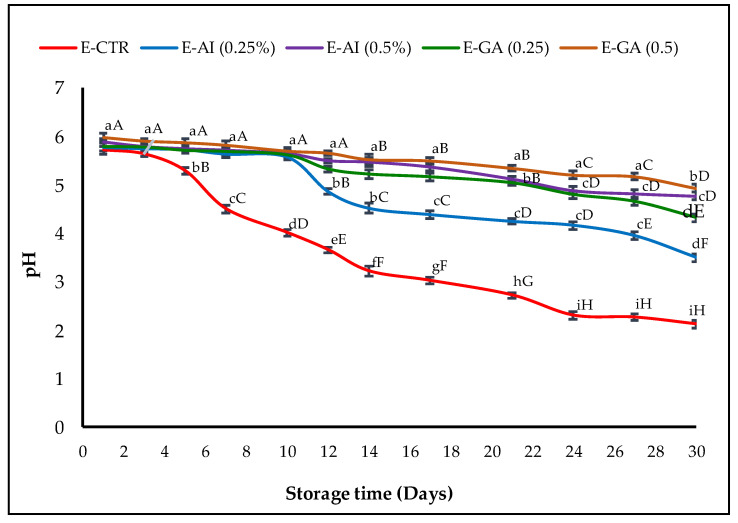
pH changes of the emulsions treated with *A. indica* leaf 50%-aqueous EtOH extract (0.25% and 0.5%, *v*/*v*) during storage. Error bars represent the standard deviation (n = 3). Lowercase letters indicate significant differences between emulsion samples on the same day at α = 0.05, and uppercase letters indicate significant differences between days of storage for the same emulsion sample α = 0.05.

Since several antioxidants are less effective in an acidic medium [53], the pH of the different emulsion samples was also measured as a potential indicator of O/W emulsion oxidation, and the results obtained are represented in Figure 2. 

All the emulsion samples started with an initial average pH value of ~5.98. The first decrease in the pH value was observed in the E-CTR sample from 5.98 to 4.50 after only seven days of storage. The E-GA (0.5%, *v*/*v*) and E-AI (0.5%, *v*/*v*) were the only samples that remained stable, with a pH of around 5 for more than 17 days before decreasing to 4.92 and 4.82, respectively, at the end of the storage time. The E-GA (0.25%, *v*/*v*) sample had a similar behaviour as the E-AI (0.25%, *v*/*v*) sample. Their pH values remained stable at around pH = 5.6 for 10 days, then decreased to pH values of 4.77 and 3.50, respectively, at the end of the storage time.

The decrease in pH is often considered a factor favouring oxidation due to the increase in the concentration of H^+^ ions in emulsion samples caused by the decrease in the effectiveness of antioxidant extracts throughout the storage period [54]. Kishk and Elsheshetawy [55] observed similar results and found that the pH values decreased in mayonnaise samples conserved with 1% and 1.25% of ginger and that the pH values of the emulsions were lower than the control sample. 

#### 2.4.3. Secondary Oxidation Products (MDA Formation)

**Figure 3:**
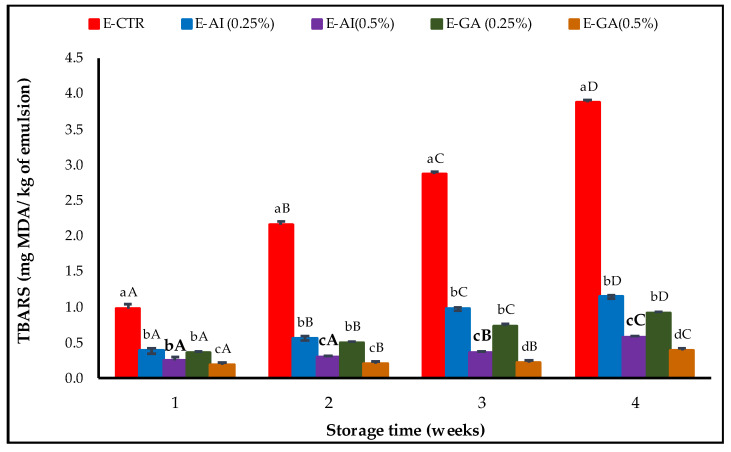
TBARS value of the emulsions incorporated with *A. indica* leaf 50%-aqueous EtOH extract (0.25% and 0.5%, *v*/*v*) during storage. Error bars represent the standard deviation (n = 3). Lowercase letters indicate significant differences between emulsion samples on the same day at α = 0.05, and uppercase letters indicate significant differences between days of storage for the same emulsion sample at α = 0.05.

MDA formation was determined by the measurement of the TBARS value, and the results obtained are shown in Figure 3.

The TBARS values obtained were significantly different between emulsion samples at α = 0.05 and increased steadily during storage. After four weeks of storage, TBARS values of the E-CTR sample were higher than the values for the rest of the samples and increased rapidly from 0.97 mg MDA/kg emulsion after the first week of storage to 3.87 mg MDA/kg emulsion at the last week. The E-AG (0.25%, *v*/*v*) and E-AI (0.25%, *v*/*v*) samples presented lower TBARS values than the E-CTR sample estimated at 0.91 and 1.14 mg MDA/kg emulsion, respectively, whereas E-GA (0.5%, *v*/*v*) and E-AI (0.5%, *v*/*v*) were the most effective samples against oxidation and presented the lowest TBARS values estimated at 0.39 and 0.57 mg MDA/kg emulsion, respectively, at the last week of the storage period. Similar findings were reported by Azman et al. [52], in which an amount of 0.5% w/w G. Lutea lyophilise was able to inhibit lipid oxidation of the oil-in-water emulsion throughout storage. MDA values for emulsions treated with 0.5% gentian powder experienced below 1.2 mg MDA/kg sample over the first 21 days and showed prominently lower values than the control (emulsion without antioxidant) up to 4 weeks. 

### 2.5. Antibacterial Activity of A. indica Leaf Extract (MIC Assay)

Table 4 exposes the results corresponding to the turbidity measurement/evaluation of penicillin and *A. indica* inoculations after 24 h of incubation. 

At concentrations ≤0.125% of both components (penicillin or plant extract), no bacterial growth inhibition was observed in all samples. On the contrary, samples with 0.25% of penicillin only showed turbidity for *E. coli* bacteria, but at higher percentages of penicillin (≤0.5%), no turbidity was observed in any sample. Concerning *A. indica* leaf extract, the concentration of 0.5 mg/mL of *A. indica* 50%-aqueous EtOH leaf extract only showed antibacterial activity against *M. luteus* and *S. paratyphi* strains. At 1 mg/mL, no bacterial growth was observed for all the microbial strains tested. 

Taking into consideration these results, the MIC of all samples were determined, and the results obtained are summarized in Table 5.

As shown, *A. indica* 50%-aqueous EtOH leaf extract was considered to have a better antibacterial activity against *S. aureus* and *E. coli* strains than penicillin, with MIC value equal to 0.50 mg/mL, whereas penicillin showed better antibacterial activity against *M. luteus* and *S. paratyphi* strains with MIC value of 0.62 mg/mL. 

### 2.6. Viability-Reducing Activity of A. indica Leaf Extract against Cancer Cell Lines

**Figure 4:**
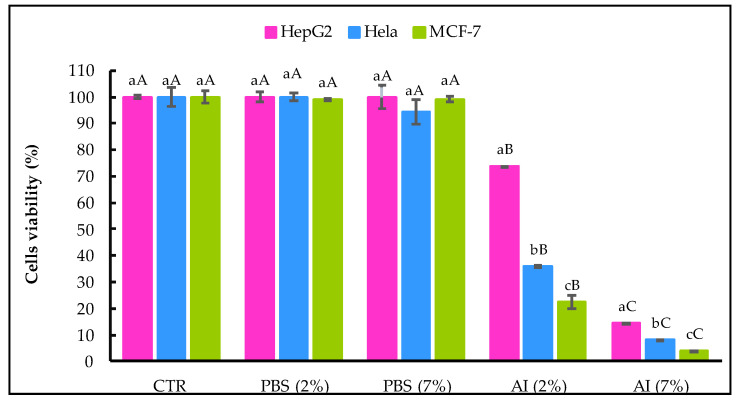
Effect of *A. indica* leaf 50%-aqueous EtOH extract (AI; 2% and 7%, *v*/*v*) on cell viability assayed 48 h after treatment. CTR, non-treated cells, PBS and control cells incubated in the presence of solvent without AI. Error bars represent the standard deviation (n = 3). Lowercase letters indicate significant differences between the three cancer cell lines with the same treatment at α = 0.05, and uppercase letters indicate significant differences between the same cancer cell line with different treatments at α = 0.05.

The viability of the HepG2, HeLa and MCF-7 cancer cell lines after being treated with *A. indica* leaf extract for 48 h was measured by MTT assay, and the results obtained are shown in Figure 4. 

No significant difference was recorded between the CTR sample and cancer cells treated with the solvent (PBS 2% and 7%, *v*/*v*). The percentage of cell viability decreased significantly (α = 0.05) with the increase in the extract concentration. *A. indica* (AI) extract at 7% (*v*/*v*) showed increased antiproliferative activity against the three cancer cell lines tested versus the AI at 2%, (*v*/*v*). At both 2% and 7%, (*v*/*v*), the AI extract was more effective in reducing the viability of cell lines derived from breast and cervix adenocarcinoma (MCF-7 and HeLa) than hepatocellular carcinoma-derived cells (HepG2). 

In HepG2 cells incubated in the presence of AI extract at 2% (*v*/*v*), the reduction in viable cells was 26.22% compared to non-treated cells, and the reduction of viable cells with AI at 7% (*v*/*v*) was 85.43%. Concerning HeLa cells, the AI extract at 2% (*v*/*v*) and 7% (*v*/*v*) decreased cancer cells by 64.01% and 85.22%, respectively. Likewise, the A.I extract at 7% (*v*/*v*) was more effective on MCF-7 cancer cells than at 2% (*v*/*v*) and reduced by 96.26% viable cells. 

Extracts of *A. indica* have been used for centuries as a natural remedy against cancer with effects attributed to the bioactive compounds present in the bark, leaves, flowers and seeds with significant anti-carcinogenic potential against adenocarcinoma and gynecological cancers, such as breast and cervical cancers [56].

Several studies proved the anticancer effect of *A. indica* extracts. For instance, Braga et al. [57] evaluated the antiproliferative activity of EtOH extracts of *A. indica* leaves collected from Brazil against MCF-7 cells using the MTT assay and found that 48 h of treatment with *A. indica* extract at a concentration of 1 µg/mL (*v*/*v*) reduced the viability of MCF-7 cells. Sharma et al. [58] evaluated the cytotoxic effect of *A. indica* extracts on MCF-7 and HeLa cells at varying concentrations and reported that *A. indica* extract got rid of more than 40% of MCF-7 cells and 60% of HeLa cells at concentrations of 350 μg/mL and 175 μg/mL, respectively. Other studies have also confirmed the sensitivity of MCF-7 and HeLa cells to *A. indica* extract [59,60]. Leaf extract of *A. indica* was also shown to induce apoptosis in HepG2 cancer cells [61].

Several studies have evaluated the capacity of extracts rich in phenolic compounds to inhibit cancer cell proliferation. For instance, Ghasemzadeh and Jaafar [62] reported that extracts of *Pandanus amaryllifolius* are rich in ferulic acid, which has a potent effect on inhibiting breast cancer cell lines in vitro. The authors of [63] identified caffeic acid in the leaves of sweet potatoes and showed that it inhibited the growth of stomach cancer, colon cancer and promyelocytic leukemia. Chlorogenic acid has also been found to have antitumor activity and potent capacity to inhibit metastasis in breast cancer cells by regulating epithelial to mesenchymal transition [64]. Chlorogenic acid is also able to inhibit autophagy and induce cell cycle arrest in human cervical carcinoma cells as well as protecting against DNA damage through the increased formation of an amino acid derivative (S-adenosyl-L-homocysteine) in MCF-7 cells [65]. Aside from phenolic acids, flavonoids may also contribute to potent anticancer effects. For instance, a recent study reported that myricetin is able to suppress cancer cell invasion and metastasis as well as to induce cell cycle arrest [66]. It must be kept in mind that phenolic compounds are only beneficial for health if the bioactive molecules are well absorbed and transported to sites affected by cancer without being metabolized into inactive molecules [67]. The importance of phenolic compounds in preventing cancer requires more in-depth research.

## 3. Materials and Methods

### 3.1. Chemicals and Standards

Epicatechin (72276); AAPH (2,2′-azobis-2-methyl-propanimidamide, dihydrochloride); acetic acid; acetonitrile; AlCl_3_ (aluminum chloride); aluminum oxide; catechin; caffeic acid (689043); chlorogenic acid (1794427); DMEM (Eagle’s Minimal Essential Medium); DMSO (dimethyl sulfoxide); DPPH (2,2-diphenyl-1-picrylhydrazyl); EtOH (ethanol); FeCl_2_ (Iron (II) chloride); ferulic acid (445858); fetal bovine serum; formazan; formic acid; FRAP reagent; gallic acid; H_2_O_2_ (hydrogen peroxide); MTT (3-(4,5-dimethylthiazol-2-yl)-2,5-diphenyl tetrazolium bromide); myricetin (5281672); p-coumaric acid (637542); quercetin (5280343); SDS (Sodium Dodecyl Sulfate); sinapinic acid (637775); sodium succinate; syringic acid (10742); TBA (Thiobarbituric acid); tween 20 and vanillin were purchased from Sigma-Aldrich Química S.A (Madrid, Spain).

Acetic acid, ammonium thiocyanate, ferric cyanide, fluorescein, Folin-Ciocalteu, HCl (hydrochloric acid), PBS (phosphate-buffered saline), quercetin and TCA (trichloroacetic acid) were acquired from Panreac Química S.L.U (Barcelona, Spain).

### 3.2. Plant Sampling and Extracts Preparation

*A. indica* leaves were collected in March 2019 from the Punjab region in the North of India, and they were accurately separated from the rest of the plant. Then, leaves were dried during one week in air under shade (23 °C), until the achievement of a constant weight. Dried leaves were crushed using an electric grinder (*KRUPS F203, Barcelona, Spain*), and the homogeneous powder obtained was then stored in amber glass bottles (to protect the samples from UV rays that could alter their contents) in a desiccator (contains lumps of silica gel and regularly calcined quicklime to absorb water vapor) at room temperature and away from the light, until the performance of the analyses. 

To prepare *A. indica* leaf extracts, one g of dry powdered leaves was mixed with 20 mL of EtOH (organic solvent) at different concentrations (50% aqueous EtOH, 80% aqueous EtOH and absolute (99.8%) EtOH). The extraction process was performed during 24 h at 4 °C, with constant stirring and using a multi-position magnetic stirrer (*Ovan, MM90E, Barcelona, Spain*). Then, the samples were centrifuged (*Orto Alresa Mod. Consul, Ajlvir, Madrid, Spain*) at 1500× *g* for 10 min, and the different supernatants were filtrated using Whatman filter paper N°1, then lyophilized for two days (*Unicryo MC2L, UniEquip Laborgerätebau & Vertr. GmbH, Martinsried, Munich, Germany*). Lyophilized samples were stored in tinted vials at 4 °C, until they were required for use.

### 3.3. Extraction Yield

The extraction yield (EY) of the different lyophilized samples was determined. The final dry weight of each sample was used to calculate the EY according to the following formula [68]:

$$\mathrm{EY}\;(\%)=\frac{W1\;}{W2}\times 100$$

where *W*_1_ represents the sample weight (mg) after the lyophilisation, and *W*_2_ represents the dry weight (mg) of the sample. 

### 3.4. Spectrophotometric Determination of Phenolic Compounds

As lyophilized 50% aqueous EtOH sample exhibited the highest EY compared with the rest of samples, this sample was chosen to perform the subsequent analyses. The absorbance was measured by a multimode micro-plate reader FLUOstar^®^ Omega (*Ortenberg, Germany*) equipped with five detection modes using an ultra-fast UV/Vis.

#### 3.4.1. Total Polyphenol Content

Total polyphenol content (TPC) was assessed following the method adapted by Gallego et al. [50], using Folin–Ciocalteu reagent. The absorbance was determined at 765 nm, and TPC was determined from a calibration curve made with gallic acid at different concentrations, which ranged between 100 and 1700 µM (R^2^ = 0.992). Results were expressed as milligram of gallic acid equivalents per gram of lyophilized sample (mg GAE/g lyophilized sample).

#### 3.4.2. Total Flavonoid Content

Total flavonoid content (TFC) was determined as described by Skowyra et al. [51] using AlCl_3_ (20 mg/mL in 5% acetic acid: MeOH). The absorbance was measured at 405 nm, and these measurements were compared to a calibration curve prepared with quercetin at different concentrations, which varied from 50 to 500 μM, (R^2^ = 0.998). Results were expressed as milligram of quercetin equivalents per gram of lyophilized sample (mg QE/g lyophilised sample).

#### 3.4.3. Total Condensed Tannin Content

Total condensed tannin content (TCTC) was determined following the method described by Julkunen-Tiitto [69], using 4% vanillin/MeOH (*v*/*v*) solution. The absorbance was measured at 550 nm. The calibration curve was prepared with catechin at different concentrations, which varied from 1 to 1000 µg/mL, (R^2^ = 0.997). Results are expressed as milligram of catechin equivalents per gram of lyophilized sample (mg CE/g lyophilized sample).

### 3.5. Antioxidant and Radical-Scavenging Activity

#### 3.5.1. Ferric-Reducing Antioxidant Power Assay

The ferric-reducing antioxidant power (FRAP) assay was estimated following the method reported by Gallego et al. [70], using the micro-plate reader at 37 °C. The absorbance was measured at a wavelength 593 nm, and FRAP values were determined from a calibration curve prepared with Trolox at different concentrations, which ranged between 3 and 20 μM, (R^2^ = 0.989). Results were expressed as milli-mole of Trolox equivalents per gram of lyophilized sample (mM TE/g lyophilized sample).

#### 3.5.2. Trolox-Equivalent Antioxidant Capacity Assay

The Trolox-equivalent antioxidant capacity (TEAC) assay was assayed according to the method described by Gallego et al. [70], using a micro-plate reader whose temperature was fixed at 30 °C. The absorbance was measured at 734 nm, and TEAC values were determined from a calibration curve made with Trolox at different concentrations, which ranged from 2 to 32 μM (R^2^ = 0.995). Results were expressed in milli-mole of Trolox equivalents per gram of lyophilized sample (mM TE/g lyophilized sample).

#### 3.5.3. Oxygen Radical Absorbance Capacity Assay

The oxygen radical absorbance capacity (ORAC) assay was performed according to the Azman et al. [71] method adapted to a micro-plate reader, at 37 °C, and using fluorescein and AAPH (0.081g/mL PBS). ORAC values were determined from a calibration curve made with Trolox at different concentrations, which ranged from 4 to 40 µM (R^2^ = 0.998). Results obtained were expressed in milli-mole of Trolox equivalents per gram of lyophilized sample (mM TE/g lyophilized sample).

#### 3.5.4. DPPH Radical-Scavenging Activity Assay

The ability of *A. indica* leaf extract to scavenge DPPH radicals was determined as described by Rahman et al. [72]. The absorbance was measured at 517 nm using the micro-plate reader, every 15 min for a total of 75 min. DPPH values were determined from a calibration curve made with Trolox at different concentrations, which ranged from 0.5 to 5 mM (R^2^ = 0.998). Results are expressed in milli-mole of Trolox equivalents per gram of lyophilized sample (mM TE/g lyophilized sample). 

### 3.6. Identification and Quantification of Phenolic Compounds by HPLC-MS

The identification and quantification of phenolic compounds in *A. indica* leaf extract were carried out using an Agilent 1200 Series HPLC-MS equipment. The equipment consists of an automatic sample injection system, two high-pressure isocratic pumps, a degasser and a chromatographic oven. The components were separated by a C18 column (100 mm × 2.1 mm, 3.5 m, Zorbax Eclipse, Agilent, Madrid, Spain) connected to a C18 precolumn (4 mm × 2 mm, Phenomenex, Torrance, CA, USA). HPLC-MS conditions were set as follows. The mobile phase was composed of Phase A (ultrapure water acidified with 0.11% formic acid) and phase B (acetonitrile acidified with 0.11% formic acid). Elution gradient corresponded to 0–2 min, 3% B; 25–27 min, 100% B; 28–38 min, 3% B, at the flow rate of 0.2 mL/min. An injection volume of 10 µL was filtered and injected into the analytical C18 column, at 30 °C (column temperature). Different commercial standards were subsequently used to identify the compounds detected by HPLC-MS. The identification of the components was confirmed by matching their retention time (RT) and the fragment ions (*m*/*z*) to those of the corresponding authentic standard compounds, in addition to the literature available.

### 3.7. Antioxidant Activity in Oil-in-Water (O/W) Food Emulsions

#### 3.7.1. Emulsions Preparation and Storage

Oil-in-water (O/W) emulsions were prepared according to: 10% purified sunflower oil (which was added drop-by-drop to an aqueous mixture) and 1% of tween-20 in a milli-Q water solution. The mixture was constantly sonicated, using an ultrasonic homogenizer (*Hielscher, UP200S, Teltow, Germany*) in an ice bath for 10 min. Sunflower oil was previously purified two times through an activated aluminium oxide (300 g of aluminium oxide was heated in the oven at 200 °C for at least 24 h to exclude all the humidity, then stored directly in the desiccator and left to cool), in absolute darkness, in order to exclude its content in tocopherols. Once prepared, this emulsion was divided evenly into different vials (5 mL), and then gallic acid (0.25% and 0.5%; *v*/*v*) and *A. indica* 50% EtOH aqueous leaf extract (0.25% and 0.5%; *v*/*v*) were added to each vial to obtain different emulsion samples at the end, including a control without antioxidant (E-CTR); an emulsion sample containing gallic acid at 0.25% (E-GA, 0.25%); an emulsion sample containing gallic acid at 0.5% (E-GA, 0.5%); emulsion containing *A. indica* leaf extract at 0.25% (E-AI, 0.25%) and emulsion containing *A. indica* leaf extract at 0.5% (E-AI, 0.5%). Emulsion samples were allowed to oxidize in an oven, at 30 ± 1 °C for 30 days, in darkness and with a constant slow agitation. 

#### 3.7.2. Primary Oxidation Reactions (Peroxide Value) and pH Measurements

Primary oxidation of emulsions was determined by the Peroxide Value (PV) method using ferric cyanide [73]. An emulsion drop (of ~10 mg of weight) was mixed with 1 mL of absolute EtOH, and the sample was mixed (with a vortex) until its complete homogenization. Then, in a plastic cuvette, 0.9 mL of each emulsion was mixed with 3.1 mL of absolute EtOH, 75 µL of FeCl_2_ (37% HCl/FeCl_2_) and 75 µL of ammonium thiocyanate (302.5 g/L). The blank contained 4 mL of absolute EtOH and 75% µL of each reactant. PV values were determined from a calibration curve obtained with H_2_O_2_ at different concentrations, which ranged from 1 to 20 mg (R² = 0.996). The absorbance was measured using a UV–vis spectrophotometer (*Zuzi spectrophotometer 4201*/*20, Auxilab, Navarra, Spain*) at 500 nm, and the results are expressed as milli-equivalents of hydroperoxides per kilogram of emulsion (meq hydroperoxides/kg emulsion).

The pH of all samples was measured daily, in triplicate, using a pH-meter (*GLP21, Criston Instruments, Barcelona, Spain*). 

#### 3.7.3. Secondary Oxidation Reactions (Malondialdehyde Content)

To measure malondialdehyde (MDA) content, 0.3 g of each emulsion was mixed with 3 mL of TBARS (Thiobarbituric Acid Reactive Substances) reactive solution (15% TCA, HCl 25 M and 0.375% TBA) in assay tubes. Tubes were then sonicated in an ultrasound water bath for 10 min at 100 °C. After cooling the tubes in ice (at 0 °C), the supernatant of each tube was removed, and the absorbance of this fraction was measured [51]. Results were expressed as milligrams of malondialdehyde per kilogram of emulsion (mg MDA/kg emulsion).

### 3.8. Antibacterial Activity 

#### 3.8.1. Microbial Strains

Four different microbial strains, which cause infective and toxic food poisoning, were provided by the “Departament de Biologia, Sanitat i Medi Ambient” of the Universitat de Barcelona (UB). These strains corresponded to: *Staphylococcus aureus* (ATCC 25923), *Micrococcus luteus* (ATCC 4698), *Escherichia coli* (ATCC 25922) and *Salmonella paratyphi* (ATCC 9150) cultures. 

#### 3.8.2. Turbidity Determination and Minimum Inhibitory Concentration Measurement

Two-fold serial dilutions of the antibiotic (penicillin, *Sigma-Aldrich Química S.A, Barcelona Spain*) and *A. indica* leaf extract were prepared. Then, 0.2 mL of each different bacterial suspension were added to each test tube, and subsequently, tubes were incubated for 24 h at 37 °C. The turbidity of penicillin and *A. indica* inoculations was evaluated in order to determine the effectiveness of the different samples in bacterial growth inhibition.

The minimum inhibitory concentration (MIC) of *A. indica* leaf extract was determined using the broth dilution method reported by Manandhar et al. [74], with some modifications. 

The MIC was calculated according to the formula described below [75]:

MIC (mg/mL) = *Lc* + *Hc*

where *Lc* represents the sample’s lowest concentration inhibiting the growth of microbial strains, and *Hc* represents the sample’s highest concentration allowing the growth of microbial strains.

### 3.9. Anti-Proliferative Activity against Cancer Cells

#### 3.9.1. Cancer Cell Lines Tested

In the current study, three different cancer cell lines were tested: MCF-7 (derived from breast adenocarcinoma), HeLa (derived from cervical adenocarcinoma) and HepG2 (derived from hepatocellular carcinoma). Cell lines were obtained from ATCC (American Type Culture Collection) nos.: CCL-2, HB-8065 and HTB-22, respectively.

#### 3.9.2. Cancer Cell Viability-Reducing Activity (MTT Assay)

Lyophilized *A. indica* leaf sample was dissolved in PBS (5 mg lyophilized extract/mLPBS solution), and then the mixture was filtered through 0.22 µm-diameter sterile filters (*Teknokroma Analítica S.A. Barcelona, Spain*). HeLa, HepG2 and MCF-7 cancer cell lines were cultured at 37 °C and maintained in a 5% CO_2_ incubator, using DMEM medium supplemented with an antibiotic solution of 100 IU/mL penicillin, 100 mg/mL streptomycin and 10% (*v*/*v*) of heat-inactivated fetal bovine serum, according to the ingredients described by Gallego et al. [76].

Cancer cell viability-reducing activity of *A. indica* leaf extract was determined using the colorimetric MTT assay, according to the method described by Kchaou et al. [77], slightly modified. In a sterile 24-well micro plate, the cancer cells (4.7 × 10 ^4^ cells /mL) were seeded in a cell growth medium for 24 h at 37 °C. Then, *A. indica* leaf extract was added at concentrations of 2% and 7% (*v*/*v*) in each well, and the cells were cultured for 48 h at 37 °C. Then, the cell growth medium was removed from the plate wells, and 120 µL of MTT reagent (2.5 mg/mL) and 420 µL of sodium succinate (6 g/mL) were added to 1 mL of fresh culture medium in each well. After 3 h of incubation at 37 °C and 5% CO_2_, the medium was removed, and formazan was suspended in a DMSO containing 0.57% acetic acid and 10% SDS. Negative controls consisted of non-treated cells and cells treated with PBS solvent. The absorbance of the different samples was measured at 570 nm in a UV spectrophotometer (*Dinko, UV2310 Barcelona, Spain*), and the viability-reducing activity of these cells was expressed in percentage (%).

### 3.10. Statistical Analysis

Statistical analyses were conducted by the Minitab statistical software (*Version 18, München, Germany*). The analysis of all the data was carried out in triplicate (n = 3), and results were expressed as means ± standard deviation (SD). Data were analysed using one-way analysis of variance (ANOVA), and Tukey’s multiple comparison test was used to determine significant differences among samples, with α = 0.05.

## 4. Conclusions

To summarize, the present work addressed the study of the antioxidant, antibacterial and antiproliferative activity of *A. indica* leaf extract from India. The antibacterial and antiproliferative activities confirmed the powerful properties of *A. indica* leaves as antibacterial and anticancer agents that can be used in phytotherapy to treat bacterial infections and cancer disease. Additionally, the antioxidant activity of *A. indica* leaf extract showed similar efficacy to that of the synthetic antioxidant by delaying food emulsion alteration and prolonging its shelf life while guaranteeing the safety of the consumer’s health. All of these results were obtained in vitro and constitute a first step in the search for the effect of natural biologically active substances from *A. indica*. Further tests will be necessary to design formulations for using the antioxidant properties of this plant in the food, pharmaceutical and cosmetic industries.

## Figures and Tables

**Table 1:** Extraction yield of *A. indica* leaves extracted in EtOH at different concentrations and fixed extraction time and temperature.

Leaf Extract	50%-Aqueous EtOH	80%-Aqueous EtOH	Absolute EtOH
EY (%)	25.14 ^a^	19.04 ^b^	12.89 ^c^

Results represent the mean of three replicates (*n* = 3) and are expressed as mean value ± SD. Different lowercase letters indicate significant differences between samples, with α = 0.05.

**Table 2:** Phenolic compounds content and radical-scavenging activity of *A. indica* leaf extracted in 50%-aqueous EtOH.

**Phenolic compound**	TPC (mg GAE/g lyophilized sample)	47.47 ± 0.03 ^a^
	TFC (mg QE/g lyophilized sample)	15.37 ± 0.12 ^b^
	TCTC (mg CE/g lyophilized sample)	11.23 ± 0.13 ^c^
**Radical-Scavenging Activity**	FRAP (mM TE/g lyophilized sample)	2.30 ± 0.01 ^a^
	TEAC (mM TE/g lyophilized sample)	1.68 ± 0.08 ^b^
	ORAC (mM TE/g lyophilized sample)	1.66 ± 0.08 ^b^
	DPPH (mM TE/g lyophilized sample)	0.37 ± 0.01 ^c^

Results represent the mean of three replicates (n = 3), and they are expressed as mean value ± SD. Different lowercase letters indicate significant differences between samples at α = 0.05.

**Table 3:** Identification and quantification of the different compounds present in *A. indica* 50% EtOH leaf extract using HPLC-MS.

Peak N°	Tentative Identification	Chemical Formula	RT (min)	Molecular Weight	Ionization Mode	Fragment Ion (*m*/*z*)		Polyphenol Class	Content *	Ref.
						Theoretical (*m*/*z*)	Observed (*m*/*z*)			
1	Chlorogenic acid	C_16_H_18_O_9_	7.29	354.3087	[M − H]^−^	353.0878	353.0880	Phenolic acids	1504.62	Std/ [30]
2	Caffeic acid	C_9_H_8_O_4_	8.34	180.1574	[M − H]^−^	179.0345	179.0350	Phenolic acids	5568.44	Std/ [30]
3	Syringic acid	C_9_H_10_O_5_	8.68	198.1727	[M − H]^−^	198.0528	197.0453	Phenolic acids	102.209	Std/ [31]
4	(-)-Epicatechin	C_15_H_14_O_6_	10.15	290.2681	[M − H]^−^	289.0717	289.0717	Flavonoids	1178.12	Std/ [32]
5	p-Coumaric acid	C_9_H_8_O_3_	11.46	164.1580	[M − H]^−^	163.0401	163.0393	Phenolic acids	5326.11	Std/ [30]
6	Ferulic acid	C_10_H_10_O_4_	13.09	194.1840	[M − H]^−^	193.0506	193.0502	Phenolic acids	418.63	Std/ [30]
7	Sinapinic acid	C_11_H_12_O_5_	13.38	224.2100	[M − H]^−^	223.0612	223.0603	Phenolic acids	7699.18	Std/ [32]
8	Myricetin	C_15_H_10_O_8_	18.27	318.2351	[M + H]^+^	319.0449	319.0427	Flavonoids	4382.05	Std/ [33]
9	Quercetin	C_15_H_10_O_7_	21.32	302.2357	[M − H]^−^	301.0354	301.0375	Flavonoids	1154.11	Std/ [33]
10	Luteolin C-hexoside I	C_21_H_20_O_11_	22.47	448.3769	[M − H]^−^	448.1006	447.0935	Flavonoids	3618	[31]
11	Cyanidin 3-O-galactoside	C_21_H_21_O_11_	23.11	449.3848	[M − H]^−^	448.1011	448.0982	Flavonoids	2845	[33]

* Expressed as microgram per g of Dry Weight (µg/g *lyophilized sample*). “Std” indicates identification of components confirmed by a standard.

**Table 4:** Turbidity determination of samples with the penicillin and *A. indica* leaf inoculations after 24 h of incubation at 37 °C.

Samples	Penicillin					*A. indica* 50%EtOH Leaf Extract					CTR (+)	CTR (−)
Cc (mg/mL)	0.062	0.125	0.25	0.5	1	0.062	0.125	0.25	0.5	1		
*S. aureus*	+	+	−	−	−	+	+	+	+	−	+	−
*M. luteus*	+	+	−	−	−	+	+	+	−	−	+	−
*S. paratyphi*	+	+	−	−	−	+	+	+	−	−	+	−
*E. coli*	+	+	+	−	−	+	+	+	+	−	+	−

(+): Turbidity, which indicates bacterial growth; (−): No turbidity, which indicates no bacterial growth.

**Table 5:** MIC values of the penicillin and *A. indica* leaf 50%-aqueous EtOH extract against the different bacterial strains.

	MIC (mg/mL)	
	Penicillin	*A. indica* 50%-Aqueous EtOH Extract
*S. aureus*	0.62	0.50
*M. luteus*	0.62	0.75
*S. paratyphi*	0.62	0.75
*E. coli*	0.75	0.50

## Data Availability

Not applicable.

## Sample Availability

Samples of Azadirachta indica are available from the authors.
